# Investigation into the Antibacterial Mechanism of Biogenic Tellurium Nanoparticles and Precursor Tellurite

**DOI:** 10.3390/ijms231911697

**Published:** 2022-10-02

**Authors:** Aiguo Tang, Qianwen Ren, Yaling Wu, Chao Wu, Yuanyuan Cheng

**Affiliations:** 1 School of Life Sciences, Anhui University, Hefei 230601, China; 2 Anhui Provincial Engineering Technology Research Center of Microorganisms and Biocatalysis, Hefei 230601, China; 3 Anhui Key Laboratory of Modern Biomanufacturing, Hefei 230601, China; 4 Anhui Province Key Laboratory of Industrial Wastewater and Environmental Treatment, Hefei 230109, China

**Keywords:** tellurite, nanoparticles, membrane damage, ROS, transcriptome

## Abstract

Antibacterial tellurium nanoparticles have the advantages of high activity and biocompatibility. Microbial synthesis of Te nanoparticles is not only a green technology but builds new ecological relationships in diverse environments. However, the antibacterial mechanism of Te nanoparticles is largely unclear. In this study, we report the bacterial synthesis of rod-shaped Te nanoparticles (BioTe) with high antibacterial activity against *Escherichia coli*. Morphology and permeability examination indicates that membrane damage is the primary reason for the antibacterial activity of BioTe, rather than ROS production and DNA damage. Moreover, a comparison of transcriptome and relative phenotypes reveals the difference in antibacterial mechanisms between BioTe and tellurite. Based on our evidence, we propose an antibacterial mode of rod-shaped BioTe, in which positively charged BioTe interact with the cell membrane through electrostatic attraction and then penetrate the membrane by using their sharp ends. In contrast, tellurite toxicity might be involved in sulfur metabolism.

## 1. Introduction

Antibacterial nanomaterials attract increasing interest for their diverse applications in clinics, medicine, consumer products, and environmental engineering. They are potential alternatives or synergists of antibiotics for when antibiotic resistance becomes a growing public health threat [1]. They are coated on consumer products or medical devices to decrease bacterial attachment and the consequent rate of human infection [2]. They are coated or fabricated on membranes to decrease the biofouling of membrane filters used for wastewater treatment [3].

Tellurium (Te) nanoparticles have become of interest due to their antibacterial, antifungal, and anticancer activity, as well as their biocompatibility [4]. The biosynthesis of Te nanoparticles by using microorganisms is a promising green nanotechnology in various areas [5]. Many prokaryotic and eukaryotic microorganisms with the ability to synthesize Te nanoparticles have been isolated from diverse environments, presumably for the detoxification of tellurite [6]. Those synthesized Te nanoparticles have various shapes, such as the needle-shaped *Halococcus salifodinae* BK3 [7], rod-shaped *Pseudomonas pseudoalcaligenes* [8], and sphere-shaped *Ochrobactrum* sp. MPV1 [9] and *Aspergillus welwitschiae* [10]. Those Te nanoparticles also show distinctive efficiency of antimicrobial activity. For example, Te nanoparticles with the needle shape from *Halococcus salifodinae* BK3 show a MIC of 2.5 μg/mL against *E. coli* [7], while those with the rod shape from *Ochrobactrum* sp. MPV1 have an MIC of 500 μg/mL against *E. coli* [9]. Besides, from the view of ecology, microbial synthesis of Te nanoparticles with antimicrobial activity in natural environments might build new ecological relationships in various niche. 

Although biosynthesis of Te nanoparticles and evaluation of their antimicrobial activity have been increasingly reported, the antimicrobial mechanism is far from clear [11,12]. In contrast to those of antibiotics, the antibacterial mechanisms of so many nanomaterials are not yet fully understood. At present, there are three antibacterial mechanisms of nanomaterials that are commonly accepted [1]. First, the interaction of nanomaterials with the cell membrane changes the morphology, permeability, and integrity of the membrane [13]. Second, nanomaterials induce the generation of reactive oxygen species (ROSs) and subsequently cause oxidative damage to biological macromolecules [14]. Third, nanoparticles cause DNA damage directly or indirectly, which commonly causes growth arrest and consequent cell death if the damage is repaired in time [15]. These mechanisms are not independent but connective with each other in many cases. 

In this work, we reported the biosynthesis of rod-shaped Te nanoparticles (BioTe) by using a tellurite-resistant bacterium we isolated previously. The BioTe showed a high activity to kill a model bacterium *Escherichia coli*. After examining all three proposed antibacterial mechanisms of nanomaterials, we found that BioTe killed cells of *E. coli* mainly by damaging the cell membrane and proposed a killing mode of BioTe by initially interacting with the membrane through electrostatic interaction and then penetrating it using their sharp ends. Moreover, we compared the antibacterial mechanisms of BioTe with tellurite which is the precursor of BioTe biosynthesis and the most common tellurium ion in diverse environments. This work will help the clinical applications of Te nanomaterials in medicine and the clinic; moreover, it will broaden our understating of their effect on environmental bacteria.

## 2. Results

### 2.1. Biosynthesis of Rod-Shaped Te Nanoparticles (BioTe)

**Figure 1:**
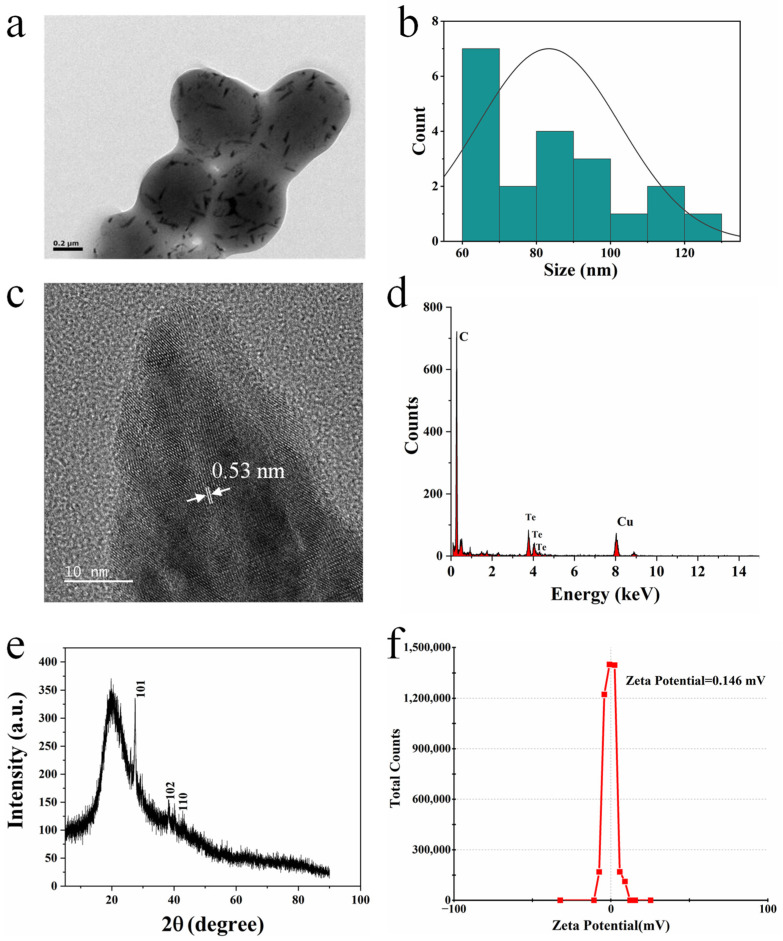
Characterization of BioTe. (**a**) Representative image of SEM of particles with cells and (**b**) distribution of the particle size determined from SEM images. (**c**) The lattice spacing of BioTe from HR-TEM image of purified particles and (**d**) Te element detection by EDS. (**e**) The XRD peak pattern of BioTe. (**f**) Charge of BioTe measured by using the zeta potential.

A tellurite-tolerant bacterium *Acinetobacter pittii* was screened and isolated from the Zhoushan saltworks in Zhejiang province. The strain was designated as *A. pittii* D120 and deposited at the China Center for Type Culture Collection as a strain CCTCC AB2020296. Growth of *A. pittii* D120 in the mineral medium containing 0.5 mM tellurite produced black materials, while no black was observed in the absence of tellurite (Figure S1). The black materials synthesized by *A. pittii* D120 were characterized. The observation made by using a scanning electron microscope (SEM) showed that the black materials were rod-shaped nanoparticles (Figure 1a) with an average size of 60–130 nm (Figure 1b). Analysis by using high-resolution transmission electron microscopy (HR-TEM) showed that the lattice spacing of the nanorods is 0.53 nm (Figure 1c), and EDS analysis detected the absorption peak of tellurium at 3.72 KeV (Figure 1d). The peak pattern of X-ray powder diffraction (XRD) was consistent with the (101) crystal planes of Te nanoparticles (Figure 1e). Biosynthesized nanoparticles usually have a natural surface modification that affects their dispersibility, stability, and activity [16]. The zeta potential of BioTe was 0.146 mV, indicating a positive charge on the surface of BioTe (Figure 1f). These results confirmed that the black materials synthesized by *A. pittii* D120 were rod-shaped BioTe with a positive charge. 

### 2.2. Antibacterial Activity of BioTe

The bacteriostatic activity of BioTe and tellurite was evaluated by the MIC at first. The MIC of BioTe and tellurite against *E. coli* BW25113 were 0.78 μg mL^−1^ and 0.36 μg mL^−1^, respectively (Table S1). Previous research shows that the MIC of BioTe nanospheres synthesized by *Ochrobactrum* sp. MPV1 is 500 μg mL^−1^ against *E. coli* strains of JM109 and ATCC 25,922 (Table S1) [9]. The MIC of BioTe synthesized in this study against *E. coli* was 714-fold lower than that in the previous report. The differences in the genotype of *E. coli* strains should not be the reason for such a gap in MIC because the MIC of BioTe we synthesized is also 0.78 μg mL^−1^ against *E. coli* JM109 (Table S1). Another possibility is that the shape and the size of nanoparticles contribute to the difference in their bactericidal activity, which is commonly reported for Ag nanoparticles [17].

**Figure 2:**
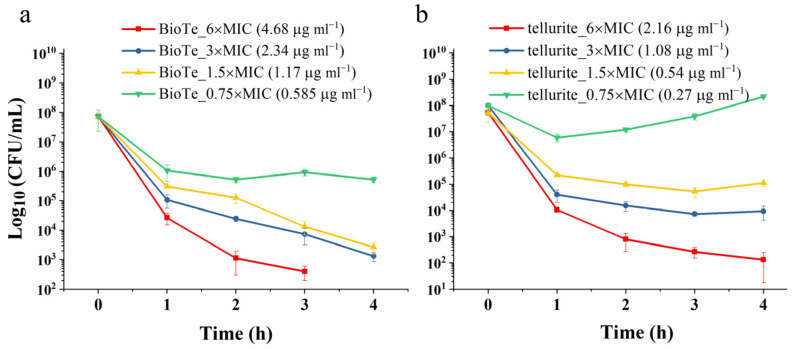
The bactericidal activity of (**a**) BioTe and (**b**) tellurite against *E. coli* BW25113. The data are the mean ± SD (*n* = 3).

The bactericidal activity of BioTe and tellurite was then examined by using the killing assay. BioTe at a concentration of 3 × MIC killed cells persistently with the extension of exposure time (Figure 2a). With a further increase of BioTe dosage to 6 × MIC, no live cells were detected for 4-h exposure (Figure 2a). In contrast, tellurite showed a different bactericidal mode from BioTe. For tellurite at a concentration of 3 × MIC, the killing rate no longer increased after treatment for more than three hours (Figure 2b). It is possible that persistent cells survived during tellurite exposure because of their lowered metabolism and tellurite import and recovered growth on a solid medium after getting rid of tellurite. These results indicated different antibacterial mechanisms between BioTe and tellurite.

### 2.3. BioTe Causes Membrane Damage of E. coli Cells

**Figure 3:**
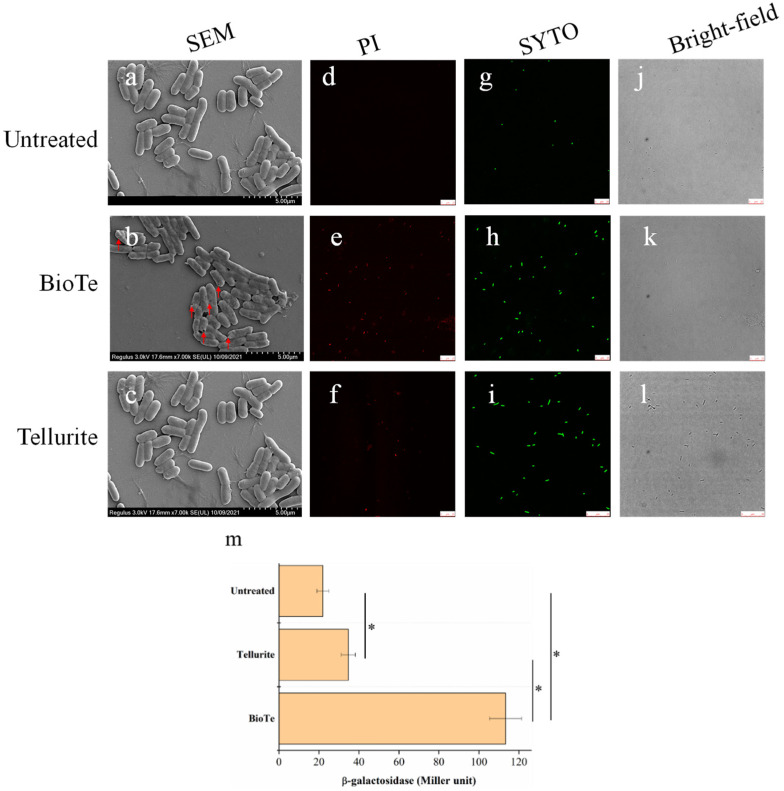
Determination of membrane integrity of *E. coli* BW25113 treated by either BioTe or tellurite. (**a**–**c**) Representative SEM images of cells in samples of untreated control, cells given BioTe treatment or those given tellurite treatment. Red arrows indicated holes in cells for treatment. (**d**–**f**) SYTO9 staining of all cells in samples of untreated control, BioTe treatment sample, or tellurite treatment sample, respectively. (**g**–**i**) PI staining of membrane-damaged cells in samples of untreated control, BioTe treatment, or tellurite treatment, respectively. (**j**–**l**) Images of cells under bright-field in respective samples. (**m**) Activity of β-galactosidase in culture supernatants from LacZ-overexpressing *E. coli* BL21(DE3), with or without treatment. Samples were treated by BioTe at 3 × MIC, or tellurite at 3 × MIC, or untreated for 1 h and then subjected to SEM observation, staining, or detection of β-galactosidase activity from LacZ. The data are the mean ± SD (*n* = 3). Stars indicated a significant difference (*p* < 0.05).

The cell membrane is a barrier that prevents entry of toxic materials into cells and maintains intracellular homeostasis. Membrane damage is one of the mechanisms of some bactericidal nanomaterials [1]. The cell morphology of *E. coli* BW25113, after being treated with either BioTe or tellurite, was observed by SEM. Compared with the smooth surface of untreated cells (Figure 3a), shrinkage and even holes on the cell surface were observed after being treated with BioTe (Figure 3b). The morphology of cells treated with tellurite was similar to that of untreated cells (Figure 3c). 

The permeability of the cell membrane was examined by transmission of extracellular chemicals into cells and leakage of an intracellular enzyme outside cells. SYTO 9 and propidium iodide (PI) are both fluorescent nucleic acid dyes. SYTO 9 can enter cells with an intact membrane, while PI can only penetrate cells with a damaged membrane; therefore, PI staining is commonly adopted as the index of membrane damage [18]. All the cells were stained by SYTO 9 in three groups of untreated control (Figure 3g,j), BioTe treatment (Figure 3h,k), and tellurite treatment (Figure 3i,l). No cells were stained by PI in the untreated control (Figure 3d). Many cells were stained by PI in the BioTe-treated culture (Figure 3e). On the contrary, a few cells were stained by PI in the tellurite-treated culture (Figure 3f). The percentage of cells stained by PI was more than 90% for BioTe treatment while less than 20% for tellurite treatment. Then, we used an *E. coli* BL21 strain overexpressing β-galactosidase to evaluate the cytoplasmic leakage caused by BioTe or tellurite. The activity of β-galactosidase leaking from cells treated with BioTe was 465% higher than that from untreated cells, indicating severe membrane damage of BioTe-treated cells (Figure 3m). The activity of β-galactosidase leaking from tellurite-treated cells was slightly higher than that from untreated control (Figure 3m), presumably due to lysis of a subset of dead cells. 

### 2.4. Involvement of ROS and DNA Damage in Antibacterial Action of BioTe

**Figure 4:**
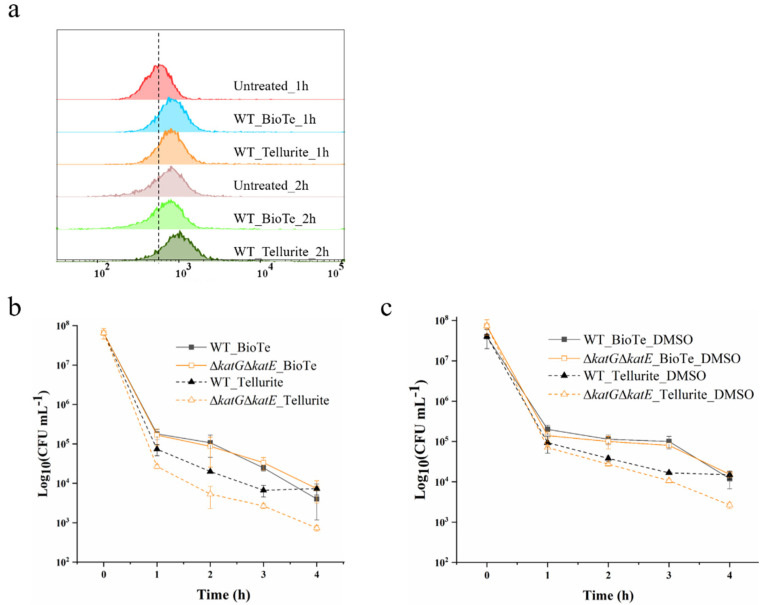
ROS induced by BioTe and tellurite and its contribution to cell death. (**a**) The ROS level in cells exposed to BioTe or tellurite. (**b**) Sensitivity of Δ*katE*Δ*katG* to the killing effect of BioTe and tellurite. (**c**) Effect of DMSO on the bactericidal properties of BioTe or tellurite. The concentration of BioTe and tellurite was 3 × MIC. The data are the mean ± SD (*n* = 3).

The induction of ROS production is known as one of the major bactericidal mechanisms of many nanoparticles [1]. Hence, we examined whether ROS was involved in the killing of *E. coli* by BioTe and tellurite. The ROS level in cells with or without treatment was measured. After being treated with BioTe or tellurite for 1 h at the concentration of 3 × MIC, cells showed a higher ROS level than untreated cells (Figure 4a). This is consistent with previous studies that tellurite causes the increase of ROS level in *E. coli* [19,20]. After treatment for 2 h, cells exposed to BioTe showed a similar level of ROS to untreated cells, and cells exposed to tellurite showed a higher ROS level. Untreated cells were incubated in an LB medium and cultivated under the same conditions as treated cells. Interestingly, we observed an increase in the ROS level in untreated cells after cultivation for 2 h (Figure 4a), presumably deriving from vigorous aerobic respiration and metabolism in the exponential growth phase [21]. The ROS level in cells treated with BioTe for 1 h did not reach higher than that in untreated cells cultivated for 2 h, while cells treated with tellurite for 2 h showed a slightly higher ROS level than the untreated cells. Such a result suggested that although BioTe induced a surge of ROS in cells, the maximal ROS level in BioTe-treated cells was not above the threshold that *E. coli* cells could handle. 

As for three naturally occurring ROS, superoxide (O_2_^−^), hydrogen peroxide (H_2_O_2_), and hydroxyl radical (•OH), *E. coli* employs the superoxide dismutase (SodA, SodB, and SodC) to catalyze the dismutation of the O_2_^-^ into O_2_ and H_2_O_2_, and catalase (KatG and KatE) to catalyze the decomposition of H_2_O_2_ into H_2_O and O_2_, while it has no protein to detoxify •OH [22]. *E. coli* mutants lacking catalase, or superoxide dismutase are more sensitive to ROS, ROS-producing materials, and chemicals [23,24,25]. Δ*katG*Δ*katE*,lacking both catalases in *E. coli* BW25113, showed an increased sensitivity to H_2_O_2_ (Figure S2). Compared to WT, Δ*katG*Δ*katE* showed similar sensitivity to BioTe while showing increased sensitivity to tellurite (Figure 4b). Given that no protein in bacteria is responsible for eliminating or capturing •OH, we examined a chemical scavenger of •OH, DMSO that is used to protect *E. coli* from killing by ROS-producing antibiotics [26]. DMSO was supplemented in the liquid medium during the processes of the killing experiment and solid medium during the following cultivation. DMSO showed no positive effect on the tolerance of both WT and Δ*katG*Δ*katE* against BioTe (Figure 4c). In contrast, DMSO increased the survival of Δ*katG*Δ*katE* exposed to tell urite (Figure 4c). Results of the killing assay indicated that ROS was involved in the antibacterial activity of tellurite rather than BioTe.

**Figure 5:**
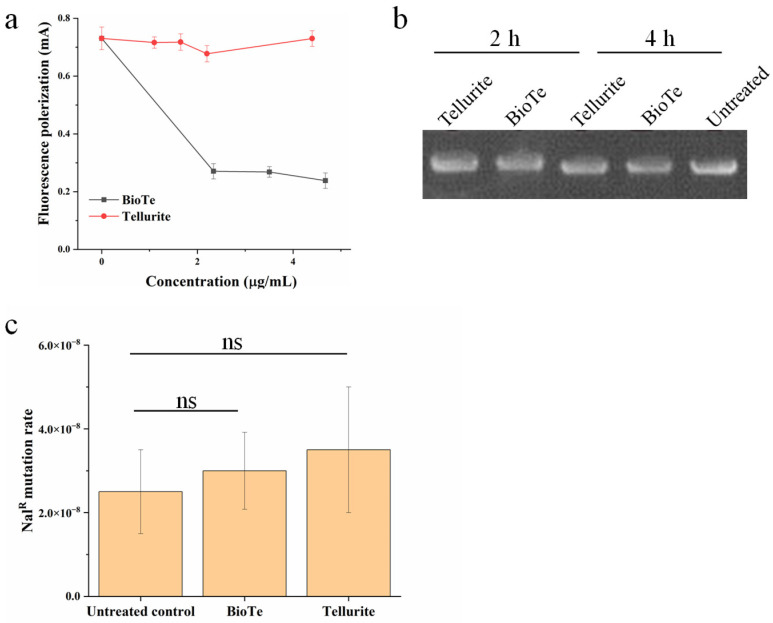
Interaction of BioTe with DNA and the consequent effect on DNA integrity. (**a**) The fluorescence polarization of FAM labeled to DNA in the presence of either BioTe or tellurite. The data are the mean ± SD (*n* = 3). (**b**) Bands of a plasmid after incubation with either BioTe or tellurite. (**c**) The mutation rate of genomic DNA of *E. coli* in vivo. Cultures of *E. coli* were treated by 3 × MIC BioTe or 3 × MIC tellurite for 3 h and then examined for DNA mutation rate. The data are the mean ± SD (*n* = 3). The ns indicates no significant difference (*p* < 0.05).

Besides ROS production, DNA damage is another primary mechanism of antibacterial nanomaterials because DNA damage immediately halts cell growth and causes cell death if it is not repaired in time [27]. We first examined the interaction of BioTe with DNA. A DNA fragment labeled by a fluorophore FAM was incubated with either BioTe or tellurite at gradually increased concentration. The polarization of fluorescence from FAM-DNA showed a dramatic decrease after FAM-DNA was incubated with BioTe (Figure 5a), indicating the binding of BioTe to FAM-DNA. In contrast, the presence of tellurite did not cause change of fluorescence polarization of FAM-DNA (Figure 5a). 

Then, we examined whether the interaction of BioTe with DNA caused DNA damage in vitro and in vivo. The plasmid is a circular molecule of double-strand DNA; nicks in one strand or breakage of double strands of a plasmid can be sensitively detected in the agarose gel. A plasmid of 6329 bp was incubated with either BioTe or tellurite and then examined by agarose gel electrophoresis. No change in the DNA band profile was observed after the plasmid was incubated with either BioTe or tellurite (Figure 5b), indicating that neither BioTe nor tellurite caused DNA damage in vitro. DNA mutation rate positively correlates with the frequency of DNA damage in bacterial cells [28]. These results suggested a possible novel toxic mechanism of BioTe, whereby their binding to DNA might hinder the transcription process rather than cause DNA damage directly. 

### 2.5. Global Response of Transcription in E. coli to Stresses of BioTe and Tellurite 

To explore the molecular mechanisms underlying the antibacterial activity of BioTe and tellurite, the transcriptome of *E. coli* BW25113 exposed to either BioTe or tellurite was compared to that of the untreated control. High dosage or long exposure time caused the death of the vast majority of cells (Figure 2), which is not suitable for transcriptome analysis because a large amount of RNA comes from dead cells and cannot reflect the physiological response of cells to either BioTe or tellurite. For this reason, a subinhibitory concentration of BioTe and tellurite was adopted that inhibits growth while not causing massive mortality for the cell population. Moreover, cells were treated for 30 min which was enough for transcriptional regulation in *E. coli* as the primary effect of the stress response, but was not too long to cause a large-scale secondary response of transcription [29]. 

**Figure 6:**
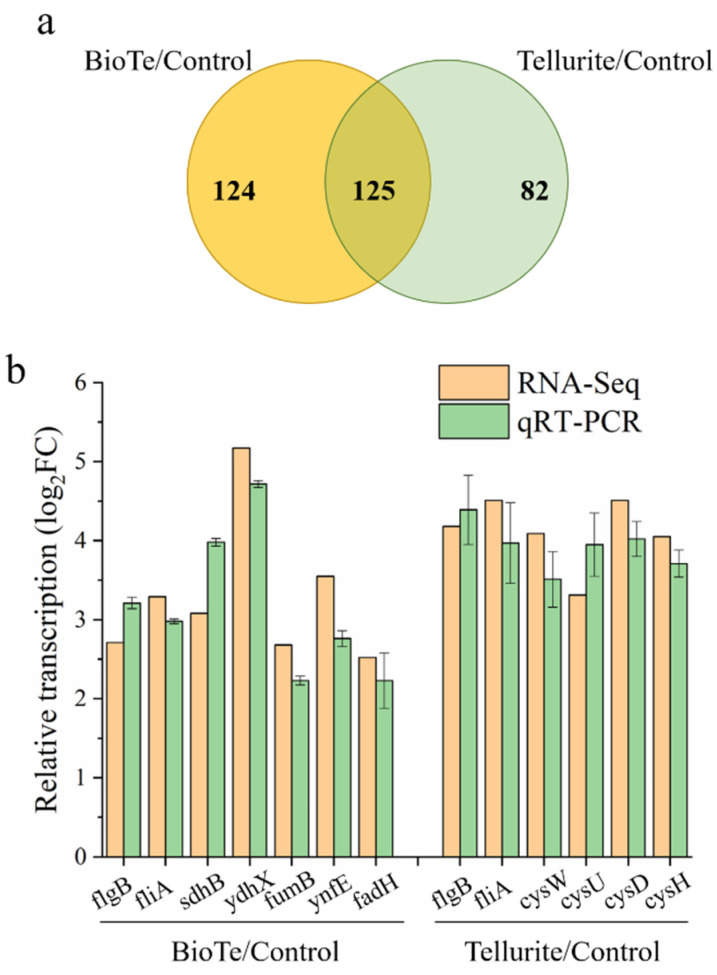
Transcriptome change of *E. coli* BW25113 treated by either BioTe or tellurite versus untreated control. (**a**) DEGs in BioTe-treated and tellurite-treated cultures compared to the untreated control, respectively. (**b**) The transcriptional change of representative genes in DEGs was examined by using qRT-PCR. The data are the mean ± SD (*n* = 3).

Compared to the untreated control, there are 249 differentially expressed genes (DEGs) in the BioTe-treated group (BioTe/control) and 207 DEGs in the tellurite-treated group (Tellurite/control) (Figure 6a). Among these DEGs, 124 genes were specifically affected in the BioTe/control and 82 genes were specifically affected in the Tellurite/control group (Figure 6a). DEGs were annotated by gene ontology (GO) enrichment analysis (Figure S3) and Kyoto Encyclopedia of Genes and Genomes (KEGG) enrichment analysis (Figure S4) to profile the global response of *E. coli* to stress of BioTe and tellurite. A large number of genes involved in flagella synthesis were strikingly upregulated in both treatment groups (Figure S3). Representative genes (*flgB*, *fliA*) were examined for their transcription in BioTe-treated and tellurite-treated cells versus untreated cells (Figure 6b). Flagella have recently been proposed as important for the resistance of *E. coli* to Ag nanoparticles, presumably by flagellin-induced aggregation of nanoparticles [30] or by enhancing flagella-mediated motility [31]. Highly toxic tellurite might act as a repellent of chemotaxis based on the function of the flagella. 

In the BioTe/Control group, transcription of several genes encoding 4Fe-4S cluster-binding proteins was specifically increased (Table S2, Figure S3). Representative genes (*sdhB*, *ydhX*, *fumB*, *ynfE*, *fadH*) were examined by using Qrt-PCR for their transcription (Figure 6b). Three possible reasons might cause upregulation of 4Fe-4S cluster-binding proteins, (i) damage of these proteins for their specific interaction with BioTe, (ii) damage of these proteins by ROS for the sensitivity of Fe–S cluster to ROS, (iii) increase in demand of these proteins for their involvement in several metabolic pathways. For example, *sdhB* encodes a subunit of the succinate dehydrogenase that is a key enzyme bridging Kreb’s (TCA) cycle and the electron transfer chain. An increased ROS level was also detected in the tellurite-treated culture, therefore damage of 4Fe-4S cluster-binding proteins by ROS contradicted with the specific upregulation in the BioT-treated sample rather than in the tellurite-treated sample. Biosynthesis of the Fe–S cluster is supposed to be enhanced if BioTe specifically damages the Fe–S cluster, However, transcription of two systems for Fe–S cluster biosynthesis (Isc and Suf) had no change on the BioTe-treated culture. Therefore, the increased transcription of these 4Fe-4S cluster-binding proteins is probably attributed to a global change in metabolism responding to the BioTe stress. 

**Figure 7:**
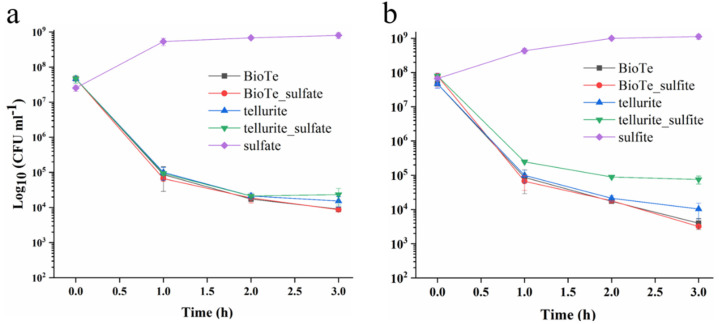
Effect of sulfate and sulfite on the tolerance of *E. coli* to BioTe and tellurite. A number of survived cells after being treated with either BioTe or tellurite both at 3 × MIC in the presence of 2.2 mg mL^−1^ sulfate (**a**) or 2.2 mg mL^−1^ sulfite (**b**). The data are the mean ± SD (*n* = 3).

In the Tellurite/control group, sulfur metabolism was specifically promoted based on KEGG enrichment analysis (Figure S4). Transcription of genes involving sulfate transport, sulfate reduction to sulfite, and further to sulfide (*cysAWUP*, *cysCND*, *cysN*, *cysHIJ*) was upregulated (Figure 6b, Table S3). Improvement in sulfur assimilation is consistent with previous research that many genes in the Cys regulon in *E. coli* are induced in the presence of potassium tellurite [32]. Sulfur deficiency causes the increased expression of sulfate transporters [33], which might occur in tellurite-exposed cells. If so, supplementation of sulfate was supposed to increase the tellurite tolerance of *E. coli*. To test this, we examined the effect of sulfate and sulfite on tellurite toxicity against *E coli*. Sulfate did not show an antagonistic effect on the tellurite toxicity (Figure 7a), while the addition of sulfite increased the survival rate of cells exposed to tellurite by about 5-fold (Figure 7b). Neither sulfate nor sulfite affected the toxicity of BioTe (Figure 7a,b). Although the underlying mechanism is unclear, these results reveal the connection between the tellurite toxicity and sulfur metabolism in *E. coli*. 

## 3. Discussion

In this study, we reported a biosynthesis method of rod-shaped Te nanoparticles (designated as BioTe) by using a tellurite-resistant bacterium. More importantly, we investigated the mechanism underlying the high antibacterial activity of BioTe against a model Gram-negative bacterium *E. coli*. The basic antibacterial actions of nanoparticles have been proposed [34]. At first, nanoparticles contact with the wall and/or the membrane of microbial cells by electrostatic attraction, van der Waals forces, or receptor–ligand interactions. This might directly cause cell death for severe membrane damage. If not, nanoparticles will further pass through the wall and/or membrane of cells, and interact with intracellular biological macromolecules including proteins, DNA, RNA, and some other important molecules such as cofactors. These interactions could cause various physiological effects including electrolyte balance disorders, protein deactivation, and changes in gene expression. Part or all these effects will induce the production of ROS that are well known for their cell toxicity. However, most of these proposed actions lack strong evidence because thorough investigations taking both biology and material science into consideration are limited. Besides themselves, nanoparticles might damage or kill cells by releasing ions, typically as Ag. 

Based on our evidence, membrane damage is the primary mechanism underlying the antibacterial activity of BioTe we synthesized. The outer membrane of Gram-negative bacteria contains lipopolysaccharides that have a large number of anionic groups and impart a negative charge to the cell membrane [35]. Such a charge characteristic makes cells of Gram-negative bacteria tend to interact with positively charged nanoparticles. Polystyrene nanoparticles with a positive charge can efficiently translocate the cell membrane while those with a negative charge show no or much less efficacy in translocation [36]. Nanosheets of graphene oxide and graphene damage the cell membrane of *E. coli* by penetrating the membrane and/or extracting phospholipids from the membrane. The sharpened edges of these nanosheets may act like blades that insert and cut the cell membrane [13]. BioTe we synthesized had a positive charge (Figure 1f) and sharp ends (Figure 1a,c). Moreover, the interaction of BioTe with cells causes the leakage of intracellular enzymes and the entering of extracellular chemicals (Figure 3). Based on these lines of evidence, we proposed an antibacterial mode of rod-shaped BioTe, in which the positive charge of BioTe attributes to the initial interaction with the cell membrane through electrostatics, and then the sharp ends of BioTe penetrate the cell membrane. Such a mode can explain the huge difference in antibacterial efficiency between rod-shaped and sphere-shaped BioTe [9]. Shape and size have been reported as determinants of the antibacterial activity of various nanoparticles. Interestingly, rod-shaped NO-releasing silica nanoparticles are more effective than sphere-shaped ones against bacterial biofilm [37]. Further research will help to reveal the generality of such a shape characteristic in the antibacterial activity of other nanoparticles.

Although the ROS level in cells after being treated with BioTe or tellurite shows a surge, it doesn’t exceed the maximal level of ROS presented in untreated cells (Figure 4a), suggesting that ROS induced by BioTe doesn’t overwhelm the ROS detoxification system in *E. coli*. The increase in ROS levels in cells during aerobic growth is not surprising because bacteria generate ROS as metabolic by-products [21]. Moreover, neither an enzyme nor a chemical of ROS scavengers shows a rescuing effect on cells exposed to BioTe (Figure 4b). These results shed light on further investigation into ROS-involved toxicity of nanoparticles against organisms under the condition of aerobic respiration. Both ROS detection and cell physiology need to be examined to draw reliable conclusions.

BioTe and tellurite show different effects on the cell membrane (Figure 3), which suggested that Te ion releasing doesn’t contribute to the toxicity of BioTe. To further reveal the difference in toxicity of BioTe and tellurite, the transcriptomes of cells exposed to either BioTe or tellurite are compared. Genes involved in sulfur metabolism are specifically upregulated by tellurite, and sulfite decreases the toxicity of tellurite to *E. coli* (Figure 6 and Figure 7). A previous study proposes that tellurite may compete with sulfite for its modifier protein to form a mixture of inter-subunit disulfide and telluric trisulfide in *Staphylococcus aureus*, a model gram-positive bacterium [38]. Our results in *E. coli* of a Gram-negative bacterium suggested that competition of tellurite with sulfite for binding to proteins might be a common toxic mechanism of tellurite against bacteria.

This study reveals that the antibacterial efficiency of rod-shaped BioTe we synthesized is attributed to the size, shape, and surface modification. Further investigation revealing the mechanism of the biosynthesis process will be helpful for better control over the shape, size, and other desired properties of the synthesized BioTe.

## 4. Materials and Methods

### 4.1. Strains and Cultivation

*A. pittii* D120 was cultured in a minimal medium (MM) (Tables S4 and S5). When needed, sodium tellurite was added into MM to a final concentration of 0.3 mM for the synthesis of BioTe. The strains of *E. coli* were cultured in Luria-Bertani medium (LB) at 37 °C with shaking at 150 rpm. When needed, kanamycin was added to 50 µg mL^−1^.

### 4.2. Biosynthesis of BioTe

*A. pittii* D120 was inoculated into MM containing sodium tellurite at a cell concentration of 1 × 10^6^ CFU mL^−1^ and incubated at 37 °C with shaking at 150 rpm under dark. The purification of BioTe was adopted from previous research with minor modifications [9]. After cultivation and synthesis for 72 h, cells and synthesized BioTe were collected by centrifugation (10,000× *g*, 10 min). The precipitate was washed three times and resuspended in deionized water. The resuspended samples were ground at 60 Hz for 6 min by using a freezing grinder JXFSTPRP-CL (Jingxin, Shanghai, China). The BioTe were separated from cell debris by centrifugation at 12,000 × *g* for 10 min. Finally, the supernatant containing BioTe was filtered through a 0.22 μm filter for sterilization. BioTe solution was stored at 4 °C before use. The concentration of BioTe solution was determined by inductively coupled plasma mass spectrometry (Thermo Fisher Scientific, Waltham, MA, USA). 

### 4.3. Characterization of BioTe

BioTe with its synthesizer *A. pittii* D120 was observed by using an SEM Regulus 8230 (Hitachi, Tokyo, Japan). Purified BioTe was analyzed by using an HR-TEM coupled with energy dispersive X-ray spectrometry JEM-F200 (JEOL, Tokyo, Japan). The particle size of BioTe was measured by using a software image J according to images of HR-TEM. To analyze the crystal structure, BioTe solution was concentrated and dried to powder by using a vacuum freeze dryer Heto PowerDry LL3000 (Thermo Fisher Scientific, Waltham, MA, USA). BioTe powder was analyzed by using an XRD Empyrean S3 (Rigaku, Tokyo, Japan). The surface charge of purified BioTe was quantified by using Zetasizer Nano ZS (Malvern Panalytical, Malvern, UK). For the zeta potential analysis, 500 μL BioTe solution was analyzed by using a Zetasizer Nano ZS. A He-Ne laser (633 nm) was used as the light source, the scattering angle was 90 degrees, and the temperature was 28 °C. The experiment was repeated three times.

### 4.4. Analysis of Antibacterial Activity 

The minimal inhibitory concentration (MIC) was determined mainly according to previous research with minor modifications [9]. Wild-type and Δ*katG*Δ*katE* derived from *E. coli* BW25113 were cultured overnight and then were diluted 100-fold and cultivated in 96-well plates containing LB and serial diluted tellurite or BioTe. These 96-well plates were incubated at 37 °C for 24 h. The MIC was determined by triplicate biological replicates in one test and repeated three times. 

The killing assay was used to evaluate the bactericidal activity of BioTe and tellurite against strains of *E. coli* BW25113. Overnight cultures were diluted 100-fold into fresh LB medium for subcultivation. When reaching OD_600_ of 0.2 (~2 × 10^8^ CFU mL^−1^), subcultures were added with BioTe or tellurite to different concentrations and were cultured continuously. At indicated time points, aliquots were removed from subcultures, washed two times, and serially diluted by using 0.9% NaCl. Diluted subcultures were spotted on LB agar plates and cultivated at 37 °C for ~24 for the development of colonies. To examine the rescuing of DMSO from ROS-caused death, DMSO was added to a final concentration of 5% in cultures and also in LB plates for cell cultivation. To examine the competition effect, sulfate or sulfite was added together with BioTe or tellurite during the killing assay to different final concentrations. 

### 4.5. Analysis of Membrane Permeability 

The staining of PI and SYTO9 was conducted by using the LIVE/DEAD BacLight bacterial viability kit (Thermo Fisher Scientific, Waltham, MA, USA). Subcultures of strains were treated by either BioTe of 2.34 µg mL^−1^ (3 × MIC) or tellurite of 1.08 µg mL^−1^ (3 × MIC) for 2 h and then stained according to the manufacturer’s instruction. After staining, cells were washed three times with and resuspended in 0.9% NaCl, and then subjected to observation under a confocal microscopy TCS SP8 DIVE (Leica Microsystem, Wetzlar, Germany).

To analyze the activity of β-galactosidase leaking from cells, the overnight culture of *E. coli* BL21(DE3) bearing a plasmid pET28a-lacZ was washed one time with and diluted into fresh LB-containing 0.1 mM isopropylthio-β-galactoside (IPTG) for a final cell concentration of OD_600_ of 0.01. When growing to OD_600_ of 0.2, cultures were added with either BioTe of 2.34 µg mL^−1^ (3 × MIC), or tellurite of 1.08 µg mL^−1^ (3 × MIC), or nothing as an untreated control. After treatment for 1 h, the cultures were centrifugated at 12,000× *g* for 30 min to remove cells, and supernatants were analyzed for the β-galactosidase activity as described previously [39].

### 4.6. Measurement of ROS 

The intracellular ROS level was detected by using 2′,7′-dichlorofluorescein diacetate (DCFH-DA) (Beyotime, Shanghai, China) which is a probe to detect ROS [40]. Overnight cultures of *E. coli* were incubated into fresh LB and grown to the early exponential phase (OD_600_ of 0.2) at 37 °C, with shaking at 150 rpm. BioTe or tellurite was added into subcultures to final concentrations of 2.34 mg/L and 1.08 mg/L, respectively, and cultivation was continued. After 2 h, DCFH-DA was added into subcultures exposed to BioTe, tellurite, or untreated control, and cultivated for a further 15 min. Stained cells in subcultures were washed with phosphate-buffered saline (0.1 M, pH 7.2) three times and resuspended in an equal volume of phosphate-buffered saline, and then subjected to analysis by using a flow cytometer CytoFLEX (Beckman Coulter, Brea, CA, USA). 

### 4.7. Analysis of Interaction with DNA and DNA Damage

The fluorescence polarization was adopted to examine the binding of BioTe to DNA [41]. A single-strand DNA (ssDNA) fragment of 27 bp was synthesized and labeled with a fluorophore carboxyfluorescein (FAM) at its 5′-end (Sangon Biotech, Shanghai, China). The other ssDNA complementary with FAM-ssDNA was also synthesized. To produce a double-strand DNA labeled with FAM (FAM-dsDNA), two fragments of ssDNA were dissolved in an annealing buffer (1 mM EDTA, 10 mM Tris, pH 8.0) and mixed at equal mole concentrations. The mixture was heated to 94 °C for 2 min and then slowly cooled down to 25 °C within 30 min. The FAM-dsDNA was examined by using electrophoresis before the assay of fluorescence polarization. The FAM-dsDNA was added to the annealing buffer containing either 2.34 µg mL^−1^ BioTe or 1.08 µg mL^−1^ tellurite at a final concentration of 8 nM. It was incubated at 37 °C for 4 h in the dark, and then fluorescence polarization was measured every 2 h by using a microplate reader SpectraMax M5 (Molecular Devices, San Jose, CA, USA).

To examine DNA damage by BioTe or tellurite in vitro, a plasmid pKD46 of 6329 bp (10 µg mL^−1^) was incubated with either 2.34 µg mL^−1^ BioTe, or 1.08 µg mL^−1^ tellurite, or ddH_2_O as an untreated control at 37 °C. At indicated time points, aliquots were removed and examined by using agarose gel electrophoresis. To examine the DNA damage in vivo, the development of resistant mutants to nalidixic acid was adopted to evaluate the mutation rate of DNA [28]. Briefly, cultures at the logarithmic phase were added with BioTe or tellurite at a concentration of 3 × MIC. After 3 h, cultures were washed three times with fresh LB. Washed cultures were spotted on LB agar plates with or without 50 μg/mL. nalidixic acid. Plates were incubated at 37 °C for 24 for the development of colonies. 

### 4.8. RNA Sequencing for Transcriptome Analysis

After being treated with either BioTe of 1/10 MIC, tellurite of 1/10 MIC, or without treatment for 30 min, cells were collected by centrifugation (12,000× *g*, 30 s, 4 °C). Four biological replicates were pooled together to comprise one sample. Cells in precipitation were resuspended with Trizol reagent (Takara Biotechnology, Beijing, China) and were vigorously shaken for thorough lysis of cells. Lyzed cells in the Trizol were stored at −80 °C before RNA extraction and RNA sequencing. Total RNA was extracted from the Trizol and further purified by using an RNA clean kit (BioTeke, Beijing, China) both according to the manufacturer’s instructions. The concentration, quality, and integrity of total RNA were determined using a spectrophotometer NanoDrop (Thermo Fisher Scientific, Waltham, MA, USA) and an Agilent 2100 bioanalyzer (Agilent Technologies, Santa Clara, CA, USA). Qualified total RNA was used to construct a sequencing library that is subsequently sequenced on a Hiseq platform (Illumina, New York, NY, USA), which was performed by Genewiz (Azenta, South Plainfield, NJ, USA). The data of RNA sequencing (RNA-Seq) have been deposited to NCBI with the accession number PRJNA863719. 

### 4.9. RNA Extraction and qRT-PCR

Cells were treated by either BioTe, tellurite, or ddH2O exactly as same as the experiment of RNA-sequencing. After treatment and collection by centrifugation, cells were lyzed using a Trizol reagent (Takara Biotechnology, Beijing, China) and total RNA was extracted according to the manufacturer’s instructions. The concentration and purity of total RNA were determined by using a spectrophotometer NanoDrop (Thermo Fisher Scientific, Waltham, MA, USA), and the integrity of total RNA was examined by RNA electrophoresis in agarose gel. 

Qualified total RNA was used for qRT-PCR. Briefly, a total RNA of 500 ng was used for cDNA synthesis by using an Evo M-MLV RT Mix Kit (Accurate Biotechnology, Changsha, China). The qRT-qPCR was conducted by using synthesized cDNA and an SYBR Premix Ex Taq kit (Takara Biotechnology, Beijing, China) on Lightcycler 96 (Roche, Mannheim, Germany). The relative expression of the target genes was calculated by using 16s rRNA as an internal reference and the 2^−^^Δ^^ΔCt^ calculation method [42]. 

## 5. Conclusions

In this study, rod-shaped Te nanoparticles, designated as BioTe, were synthesized using a tellurite tolerant bacterium *Acinetobacter pittii* D120. This rod-shaped BioTe showed bactericidal activity against the model Gram-negative bacterium *E. coli*. The MIC of BioTe was more than 700 folds lower than that of previously reported sphere-shaped Te nanoparticles. In addition, BioTe showed more persistent bactericidal activity than tellurite. Examination of the transcriptome reveals the similarity and difference in bactericidal mechanism between BioTe and tellurite.

The bactericidal mechanism of BioTe was examined from three aspects, membrane damage, ROS induction, and DNA damage. First, BioTe caused severe leaking of intracellular enzymes and entrance of a membrane-impermeable DNA dye, which indicated obvious membrane damage. Second, BioTe caused an increase in intracellular ROS levels, while chemical and biological quenchers of ROS did not rescue cells of *E. coli* from BioTe toxicity. Moreover, the ROS level caused by BioTe did not overwhelm the ROS detoxification capability of *E. coli*. Therefore, the bactericidal activity of BioTe did not attribute to ROS induction. Third, BioTe was able to bind DNA in vitro, however, did not cause detectable DNA damage in vitro and in vivo. Based on these results, we proposed a shape-determined bactericidal mechanism of BioTe, which heavily depended on the membrane damage of *E. coli* cells. 

## Data Availability

The data of RNA sequencing (RNA-Seq) have been deposited to NCBI with the accession number of PRJNA863719.

## Supplementary Materials

The following supporting information can be downloaded at: https://www.mdpi.com/article/10.3390/ijms231911697/s1, References [9,43] are cited in the supplementary materials.
