# Supplementary material for: Investigation into the Antibacterial Mechanism of Biogenic Tellurium Nanoparticles and Precursor Tellurite
Source: Int J Mol Sci. 2022 Oct 2;23(19):11697. doi: 10.3390/ijms231911697 (PMC9569536; doi:10.3390/ijms231911697)
Supplement: Supplementary file 1 [file ijms-23-11697-s001.zip › ijms-1918473-supplementary.pdf]

Table S1. MIC of BioTe and tellurite against bacteria.

Materials	Forms	Examined strains	MIC (mg/L)	References
BioTe	Rods	*Escherichia coli* BW25113	0.78	In this study
		E. coli JM109	0.78	
	Sphere	*E. coli* JM109	500	[1]
		E. coli ATCC 25922	500	
	Rods	*Staphylococcus aureus*	250	[2]
		Pseudomonas aeruginosa	125	
TeO32-	-	*E. coli* BW25113	0.36	In this study

Table S2. Discussed genes that differentially expressed genes in *E. coli* exposed to BioTe.

Locus tag	Gene name	Molecular Function	LogFC[a]
BW25113_0724	*sdhB*	2 iron, 2 sulfur cluster binding; 3 iron, 4 sulfur cluster binding; 4 iron, 4 sulfur cluster binding; electron transfer activity; iron–sulfur cluster binding;	3.084702
BW25113_1587	*ynfE*	4 iron, 4 sulfur cluster binding; electron transfer activity	3.550983
BW25113_1588	*ynfF*		3.252602
BW25113_1589	*ynfG*	4 iron, 4 sulfur cluster binding; metal ion binding; electron transfer protein	2.975999
BW25113_1590	*ynfH*	oxidoreductase activity; membrane anchor protein	2.626747
BW25113_1671	*ydhX*	4 iron, 4 sulfur cluster binding; membrane protein	5.167056
BW25113_1673	*ydhV*	4 iron, 4 sulfur cluster binding; electron transfer activity; oxidoreductase activity	4.092291
BW25113_2880	*ygfM*	FAD binding; oxidoreductase activity	3.311295
BW25113_3115	*tdcD*	ATP binding; metal ion binding; propionate kinase activity	3.455344
BW25113_3116	*tdcC*	serine transmembrane transporter activity; threonine efflux transmembrane transporter activity	2.438295
BW25113_4122	*fumB*	4 iron, 4 sulfur cluster binding; D (-)-tartrate dehydratase activity; fumarate hydratase activity; membrane protein	2.675096
BW25113_1729	*ydjN*	sulfur amino acid transmembrane transporter activity;	-0.70735
BW25113_2012	*yeeD*	putative sulfur transferase activity	-0.31561

[a]. FC indicates fold change of transcription in *E. coli* BW25113 exposed to BioTe versus the untreated control.

Table S3. Discussed genes that differentially expressed genes in *E. coli* exposed to tellurite.

Locus tag	Gene	Molecular function	LogFC[a]
BW25113_1729	*ydjN*	sulfur amino acid transmembrane transporter activity;	3.550983
BW25113_2012	*yeeD*	putative sulfurtransferase activity	3.252602
BW25113_2013	*yeeE*	putative sulfurtransferase activity	2.975999
BW25113_2141	*yohJ*	putative sulfurtransferase activity	2.626747
BW25113_2142	*yohK*	putative sulfurtransferase activity	3.627047
BW25113_2423	*cysW*	ATPase-coupled sulfate transmembrane transporter activity	5.167056
BW25113_2424	*cysU*	ATPase-coupled sulfate transmembrane transporter activity	3.209431
BW25113_2425	*cysP*	ATPase-coupled sulfate transmembrane transporter activity, sulfate binding, thiosulfate binding	4.092291
BW25113_2752	*cysD*	ATP binding, sulfate adenylyltransferase (ATP) activity	3.311295
BW25113_2764	*cysJ*	sulfite reductase (NADPH) activity	3.455344
BW25113_3116	*tdcC*	serine transmembrane transporter activity; threonine efflux transmembrane transporter activity	-0.41135
BW25113_4122	*fumB*	4 iron, 4 sulfur cluster binding; D (-)-tartrate dehydratase activity; fumarate hydratase activity; membrane protein	-1.40229

[a]. FC indicates fold change of transcription in E. coli BW25113 exposed to Na_2TeO^3 versus the untreated control.

Table S4. Formulation of the minimal medium. An additional 5 mL of trace elements was added.

Chemical formulas	Concentration (g/L)
K_2HPO_4	0.225
KH_2PO_4	0.225
NaCl	0.46
$(NH_4)_2SO_4$	0.0255
$MgSO_4 \cdot 7H_2O$	0.024
$C_8H_{18}N_2O_4S$	4.766

Table S5. Formulation of trace element used in the minimal medium.

Chemical formulas	Concentration (g/L)
$N(CH_2COOH)_3$	1.5
$MgSO_4$	3.0
$CaCl_2{\cdot}2H_2O$	1.0
NaCl	1.0
$MnSO_4$	0.5
$ZnSO_4 \cdot 7H_2O$	0.18
$FeSO_4{\cdot}7H_2O$	0.1
$CoSO_4{\cdot}7H_2O$	0.18
$NiCl2{\cdot}6H_2O$	0.025
$KAl(SO_4)_2{\cdot}12H_2O$	0.02
$CuSO_4{\cdot}5H_2O$	0.01
H_3BO_3	0.01
$H_4MoNa_2O_6$	0.01
$H_{10}Na_2O_8Se$	0.0003

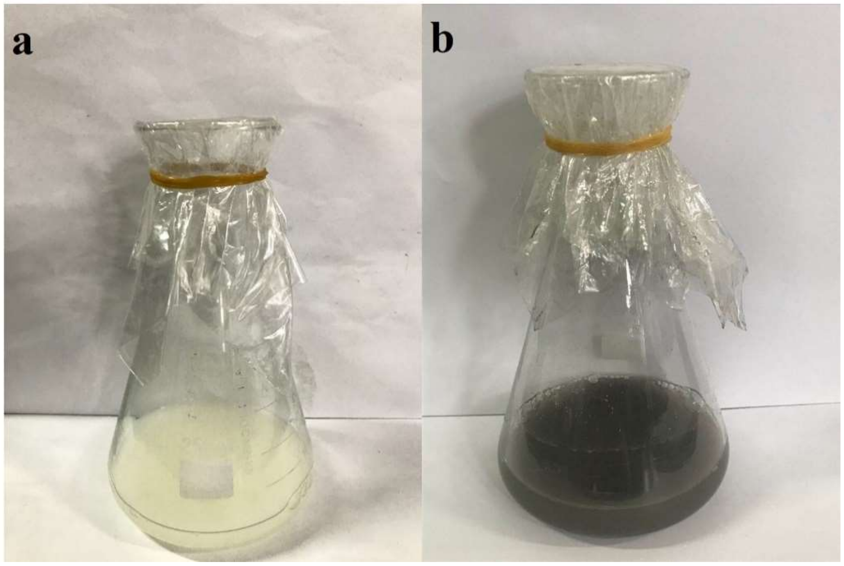

Figure S1. Synthesis of BioTe by *Acinetobacter Pittii* D120. The color of cultures after they were cultivated in media (a) without or (b) with 0.5 mM tellurite.

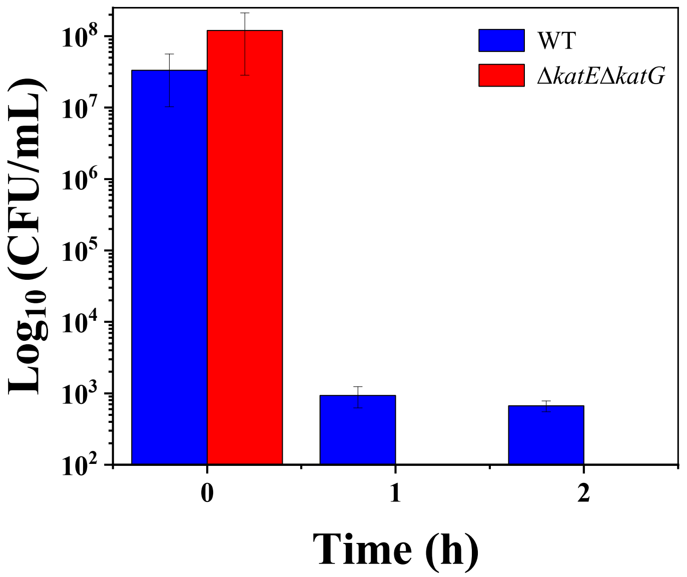

Figure S2. Susceptibility of the wild-type strain and a *ΔkatEΔkatG* mutant of *E. coli* BW25113 to H_2O_2. Cultures were exposed to 10 mM H_2O_2. No CFU was observed for *ΔkatEΔkatG* after being treated for 1 h and 2 h. The data are the mean ± SD (n = 3).

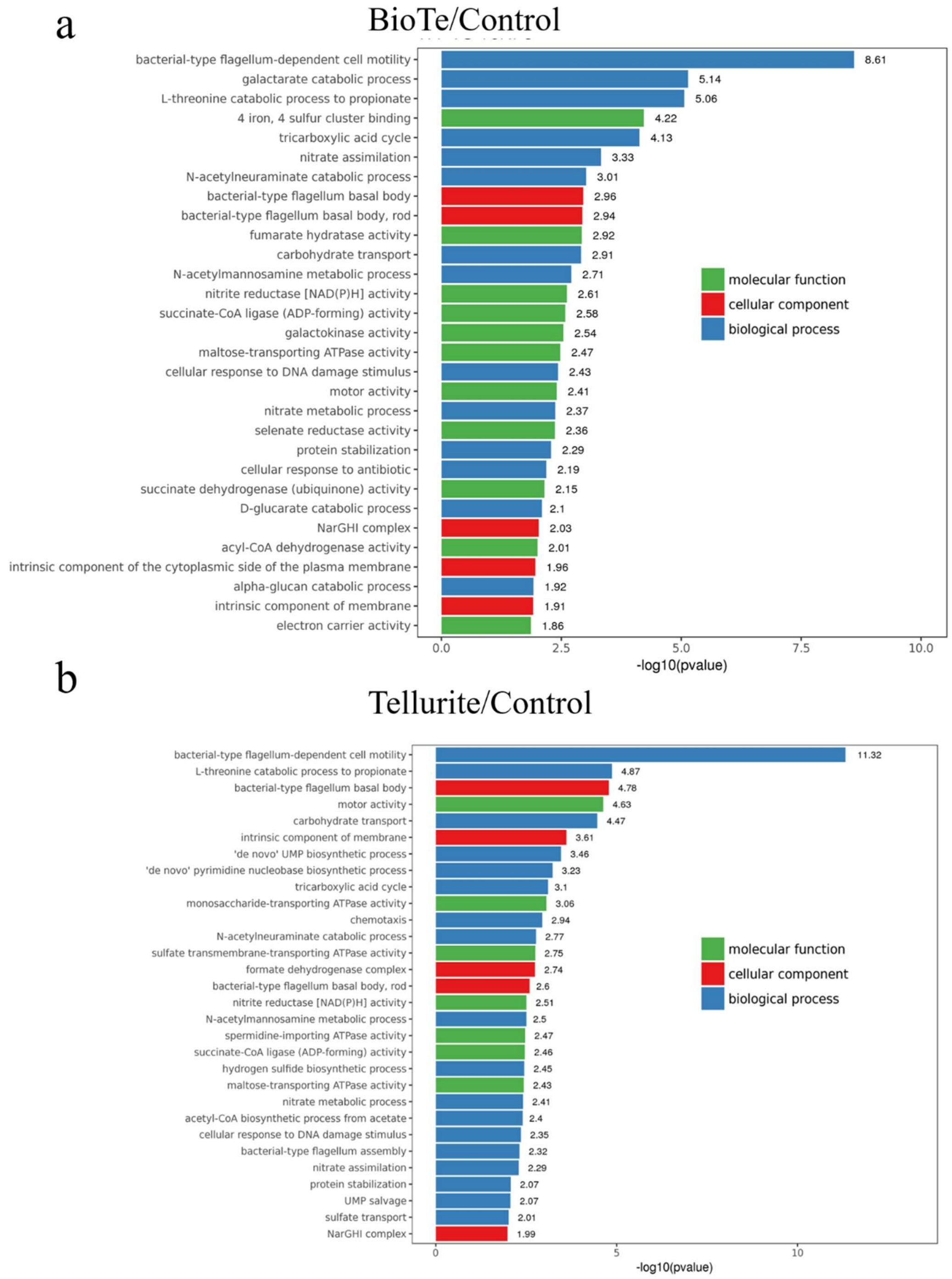

Figure S3. GO analysis report of differentially regulated genes in (a) BioTe-treated cultures and (b) in tellurite-treated cultures, compared to untreated cultures. The GO categories cover three domains: biological process (BP), cellular component (CC) and molecular function (MF).

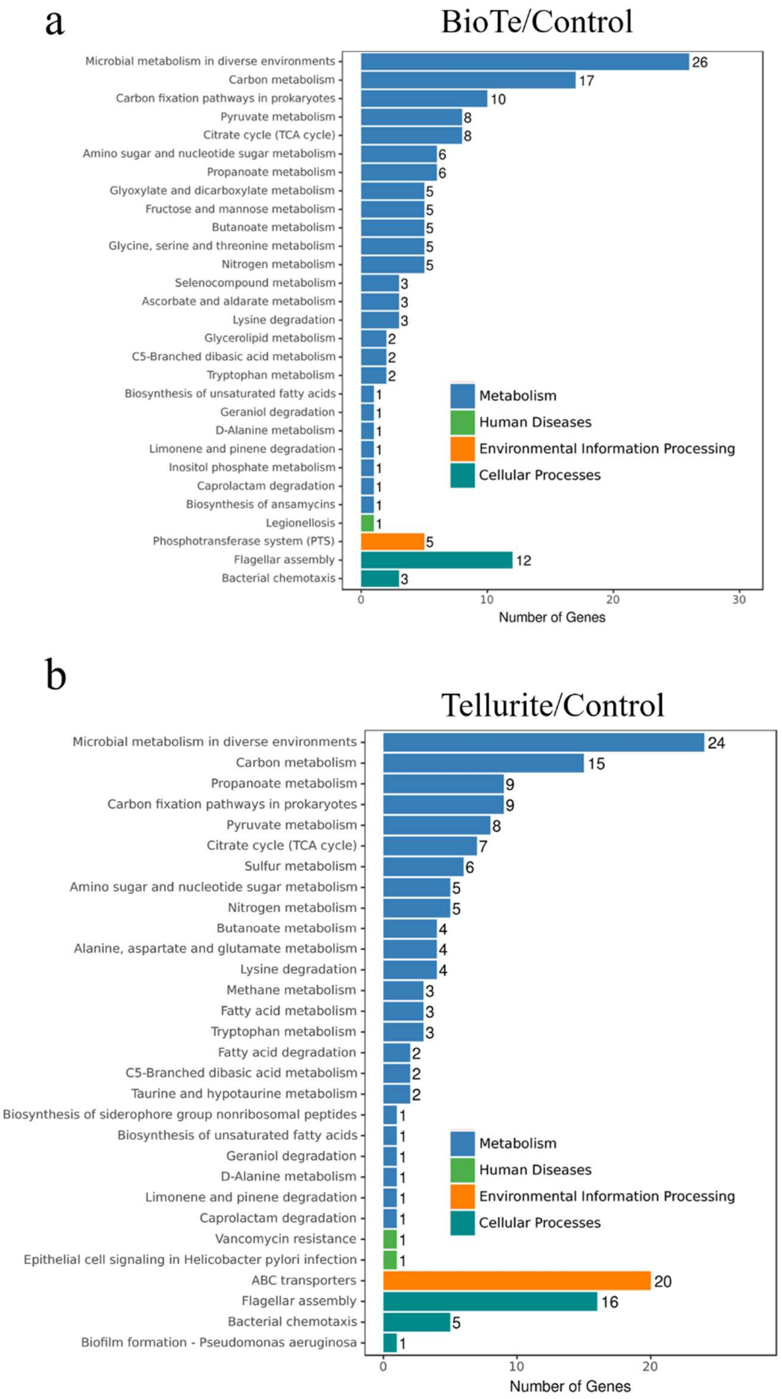

Figure S4. Pathway analysis report of differentially regulated genes in (a) BioTe-treated cultures and (b) in tellurite-treated cultures, compared to untreated cultures.

Reference

1. Zonaro, E.; Lampis, S.; Turner, R.J.; Qazi, S.J.; Vallini, G. Biogenic selenium and tellurium nanoparticles synthesized by environmental microbial isolates efficaciously inhibit bacterial planktonic cultures and biofilms. Front Microbiol 2015, 6, 584, doi:10.3389/fmicb.2015.00584.
2. Zare, B.; Faramarzi, M.A.; Sepehrizadeh, Z.; Shakibaie, M.; Rezaie, S.; Shahverdi, A.R. Biosynthesis and recovery of rod-shaped tellurium nanoparticles and their bactericidal activities. Materials Research Bulletin 2012, 47, 3719-3725, doi:10.1016/j.materresbull.2012.06.034.
